# Analgesic effects of bulleyaconitine A: new advances in research from ion channel targets to clinical translation

**DOI:** 10.3389/fphar.2025.1733973

**Published:** 2026-01-15

**Authors:** Zili Yin, Xinlian Song, Changcheng Zhu, Anguo Hou, Rong Chen

**Affiliations:** 1 Yunnan Key Laboratory of Dai and Yi Medicines, Yunnan University of Chinese Medicine, Kunming, Yunnan, China; 2 Kunming Medical University Haiyuan College, Kunming, Yunnan, China

**Keywords:** analgesic mechanism, bulleyaconitine A, chronic pain, clinical application, novel drug delivery system, separation of toxicity and efficacy

## Abstract

Chronic pain represents a significant global health concern, posing a severe threat to human wellbeing and affecting up to 20% of the adult population. Bulleyaconitine A (BLA), a diterpenoid alkaloid derived from plants of the Aconitum genus with in the Ranunculaceae family, demonstrates remarkable analgesic properties with a low potential for addiction, thus broad clinical application prospects compared to opioids. Extensive research has elucidated multiple pharmacological mechanisms underlying the analgesic effects of BLA, including state-dependent blockade of voltage-gated sodium channels, activation of the κ-opioid receptor pathway in spinal microglia, and anti-inflammatory immunomodulatory effects through inhibition of the NF-κB pathway. These mechanisms have been validated in various pain models, including neuropathic pain, cancer pain, rheumatoid arthritis pain, and visceral pain. However, BLA still faces pharmacokinetic challenges in clinical translation, including a narrow therapeutic window, low bioavailability, and potential neurotoxicity. In recent years, the development of novel drug delivery systems, structural modifications targeting the C-8 and C-14 sites to separate toxicity from efficacy, and advances in artificial intelligence-assisted drug design have provided effective solutions to overcome these limitations. Emerging research suggests that BLA has potential new indications in areas such as visceral hypersensitivity, irritable bowel syndrome, and pain-related anxiety disorders. This article provides a comprehensive review of the ion channel targets, central and peripheral mechanisms of action, pharmacokinetic characteristics, innovative drug delivery strategies, structural optimization pathways, and current clinical application of BLA. It aims to offer valuable references for the further development and rational clinical application of BLA as a non-opioid analgesic with multi-target therapeutic potential.

## 1 Introduction

Pain is defined as a physical or emotional discomfort associated with actual or potential tissue damage (Malik, 2020). Based on its characteristics, pain is categorized into chronic pain, neuropathic pain, and nociceptive pain (Kataria et al., 2024). Patients frequently experience severe sleep disturbances, anxiety, and depression, which significantly impair their quality of life (Rao et al., 2024). According to data from the World Health Organization (WHO), up to 20% of adults worldwide experience pain, highlighting its status as a significant public health concern (Kosek et al., 2016).

Currently, the commonly employed clinical methods for pain management encompass both pharmacological and non-pharmacological treatments. Pharmacological interventions include opioids and nonsteroidal anti-inflammatory drugs (NSAIDs), such as aspirin, while non-pharmacological approaches comprise cognitive behavioral therapy, physical therapy, and interventional procedures like nerve blocks (He et al., 2024). Nevertheless, existing therapies present several limitations, including the potential for drug abuse, addiction, gastrointestinal adverse reactions, and risks associated with invasive procedures (Kaye et al., 2020). The structural diversity inherent in natural products underpins their varied and unique biological activities. Consequently, in the pursuit of treatments with reduced side effects, there has been a growing emphasis on traditional herbal medicines and their constituent monomeric compounds.

BLA was first isolated from the Ranunculaceae plant Aconitum bulleyanum (Aconitum bulleyanum Diels) by Wang Fengpeng and Fang Qicheng (Wang, 1981) in 1981. It is a significant component of the Aconitum genus. BLA is a diterpenoid alkaloid that has been included in the *Chinese Pharmacopoeia* (2020 edition) due to its biosafety and has been approved for clinical application in China. For its extraction and purification, ethanol or methanol is typically employed as the primary solvent, often in conjunction with ultrasonic treatment to enhance extraction efficiency. The crude extract undergoes acid-base refinement: it is initially dissolved in dilute hydrochloric acid (0.5%–1%) to solubilize the alkaloid, followed by pH adjustment to 9-10 using ammonia water to precipitate the free base, which is subsequently extracted with chloroform. Purification is achieved through silica gel column chromatography employing a chloroform-methanol gradient elution system, and the final medicinal-grade pure product is obtained via ethanol recrystallization (Fu et al., 2024).

Clinically, BLA was formally incorporated into the *Expert Consensus on Diagnosis and Treatment of Neuropathic Pain* in 2013 (Xu and Yao, 2023), and is also referened in the latest *Chinese Guidelines for the Assessment and Management of Neuropathic Pain* (2024 edition) as well as the *Chinese Guidelines for the Diagnosis and Treatment of Chronic Post-Traumatic Pain* (2023 edition)(Jia et al., 2025). BLA has demonstrated excellent anti-inflammatory and immunomodulatory effects, acting on multiple sites of pain perception and exhibiting dual pharmacological actions of central and peripheral analgesia. It has shown good efficacy in treating various types of chronic pain, including cancer pain (Ling et al., 2007), neuropathic pain (Yang et al., 2013), and rheumatoid arthritis pain (Tang et al., 1986). Reports indicate that BLA, compared to opioids, is non-addictive, and has fewer gastrointestinal adverse effects than nonsteroidal anti-inflammatory drugs like aspirin (Xiao et al., 2021), with its analgesic effect being stronger than that of morphine and aspirin (Tang et al., 1986).

Despite the promising potential of BLA, several challenges remain in its clinical translation, including a narrow therapeutic window, low bioavailability, cardiovascular toxicity (such as palpitations and arrhythmias),and potential neurotoxicity, which limit its broader clinical application. In recent years, advancements in drug delivery systems, structural modifications, and artificial intelligence-assisted drug design have led to a renewed recognition of BLA’s potential.

This article will review the mechanism of action, pharmacokinetics, and drug delivery strategies of BLA, as well as progress in structural modifications and toxicity separation.

**FIGURE 1:**
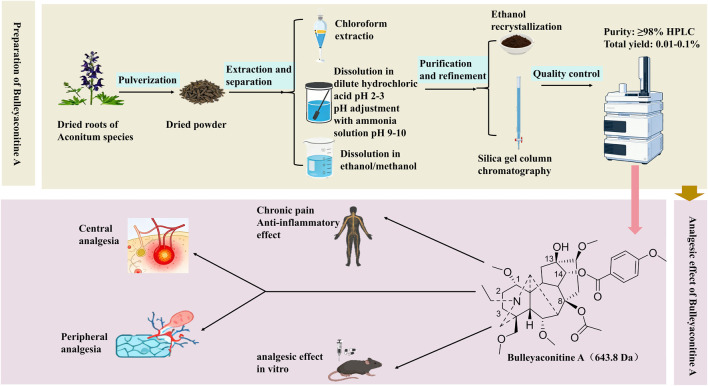
Preparation process and pharmacological effects of BLA.

Additionally, it will discuss clinical applications and prospects for new indications, proposing future development directions based on the latest research to provide references for its further development and rational utilization (Figure 1).

## 2 BLA from single target to multi-pathway regulation

### 2.1 Ion channel targets

**FIGURE 2:**
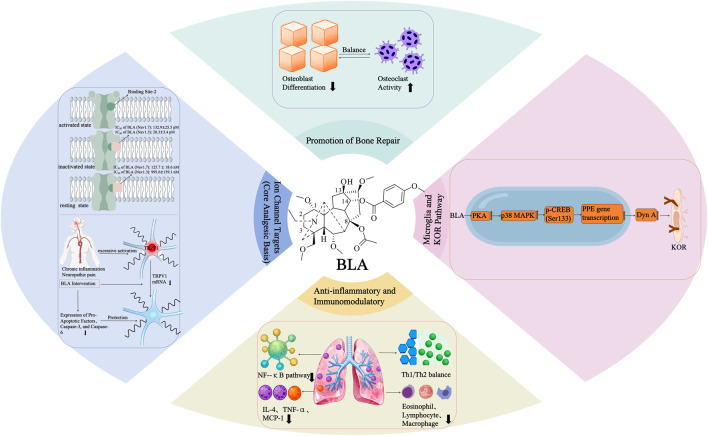
BLA: From single target to regulation of multiple pathways.

Ion channels are pivotal targets of BLA in its analgesic mechanism, encompassing voltage-gated sodium channels (VGSCs), transient receptor potential channels (TRP), and calcium channels, among others. These channels are essential for peripheral nociception and central pain transmission. Numerous studies have demonstrated that BLA induces analgesia by modulating these channels, thereby providing significant insights into its molecular mechanism (Figure 2).

#### 2.1.1 Sodium channel state-dependent mechanism

Voltage-gated sodium channels (Nav) are critical targets for the treatment of various neurological disorders (Catterall et al., 2020). They primarily exist in three functional states: resting, activated, and inactivated (Catterall, 2014). BLA preferentially binds to inactivated channels during high-frequency firing or sustained depolarization, demonstrating a state-dependent blockade with minimal impact on resting neurons (Wang et al., 2007).

Wang et al. (2008) demonstrated through patch-clamp studies that BLA exhibits minimal inhibition on Nav1.7 and Nav1.8 in the resting state, yet significantly enhances blockade in the inactivated state. Further research by Xie et al. (Xie et al., 2018a) revealed that the IC_50_ for the inactivated state of Nav1.7 (132.9 ± 25.5 pM) is approximately 946 times lower than that for the resting state (125.7 ± 18.6 nM). Additionally, the IC_50_ for the inactivated state of Nav1.3 (20.3 ± 3.4 pM) is approximately 49,000 times lower than that for the resting state (995.6 ± 139.1 nM).

This property enables BLA to specifically inhibit hyperactivated neurons in neuropathic pain (Cummins et al., 2007) while minimally impacting normal nerve conduction (Ruiz and Kraus, 2015), thereby optimizing the preservation of normal nerve conduction function.

Recently, Xiao et al. (2025) employed cryo-electron microscopy to elucidate the structure of the BLA-human Nav1.3 complex. Their findings revealed that the complex binds to the site-2 position of the central pore of the channel, stabilizing the open conformation of the DI–DII S6 helix while partially obstructing the ion channel. This dual effect elucidates the mechanism by which the complex promotes channel opening while simultaneously inhibiting ionic current. This structural study lays a crucial foundation for the development of novel Nav modulators characterized by enhanced selectivity and an expanded therapeutic window.

#### 2.1.2 Regulatory effects on TRPV1

The transient receptor potential vanilloid type 1 (TRPV1) is a crucial ion channel in nociceptive neurons that plays a significant role in the perception of heat, acidity, and inflammatory factors (Tominaga and Caterina, 2004). In conditions of chronic inflammation and neuropathic pain, excessive activation of TRPV1 results in hyperalgesia and spontaneous pain (Cortright and Szallasi, 2009). Research has demonstrated (Wei et al., 2020) that BLA significantly inhibits both mRNA and protein expression of the TRPV1 receptor in spinal cord tissue, exhibiting a clear dose-dependent effect. Furthermore, BLA reduces the expression levels of pro-apoptotic factors such as Fas, Caspase-3, and Caspase-6, thereby exerting a neuroprotective effect. This indicates that BLA not only operates through the classical sodium channel mechanism but may also inhibit neuronal apoptosis by downregulating the sensitivity of both central and peripheral TRPV1 receptors, consequently alleviating neuropathic pain (Table 1).

**TABLE 1:** Ion channel targets of the BLA.

Channel category	Specific pathway	Core mechanism of BLA	Physiopathological significance
VGSCs	Nav1.3 Nav1.7 Nav1.8	① State-dependent block: preferential binding to inactivated channels ② Bound to the central pore channel site-2 ③ Dual regulation: stabilizing the open conformation of the S6 helix and partially obstructing the channel	Selectively inhibiting hyperactive neurons in neuropathic pain while preserving normal nerve conduction function
TRP	TRPV1	① Downregulation of mRNA and protein expression ② Reduce the expression of Fas and Caspase-3/6, and protect neurons	Alleviate chronic inflammation and neuropathic pain, reduce hyperalgesia and spontaneous pain

### 2.2 Central microglia and the κ-opioid receptor pathway

#### 2.2.1 The mechanism of action of central microglia

The analgesic effect of BLA in the central nervous system is not solely dependent on ion channels; it also plays a unique role by modulating microglia in the spinal dorsal horn (Huang et al., 2016; Li et al., 2016). In the context of chronic pain pathology, microglia in the spinal dorsal horn facilitate inflammatory responses and neuronal sensitization, which in turn exacerbates the transmission of pain signals (Clark et al., 2013; Tsuda et al., 2003).

Traditional views suggest that microglial activation is a significant driving factor in the chronicity of pain. However, Huang et al. (2016) confirmed that BLA-specific stimulation induces spinal microglia to express the endogenous κ-opioid receptor (KOR) agonist dynorphin A (Dyn A) (Grace et al., 2014), which inhibits the release of excitatory neurotransmitters and enhances the activity of inhibitory neurons, thereby producing an analgesic effect (Ji et al., 2018). Unlike traditional anti-inflammatory drugs, BLA does not inhibit peripheral pro-inflammatory factors, demonstrating selective regulatory characteristics.

Li et al. (2017) further elucidated the molecular signaling mechanisms by which the BLA regulates dynorphin A expression in microglia. The study demonstrated that the BLA first activates protein kinase A (PKA), which subsequently activates p38 mitogen-activated protein kinase (MAPK). The activated p38 MAPK then phosphorylates cAMP response element-binding protein (CREB) at the Ser133 site, thereby promoting the transcription of the prodynorphin gene (PPE).

This unique molecular mechanism holds significant therapeutic implications. BLA selectively induces microglia to release endogenous dynorphin A by activating the PKA-p38 MAPK-CREB pathway, rather than suppressing pro-inflammatory responses. Compared to traditional strategies for inhibiting microglia, this approach of activating the endogenous analgesic system mitigates the risk of excessive immune suppression.

Dynorphin A, an endogenous κ-opioid receptor agonist, exerts potent analgesic effects at the spinal level. Compared to traditional μ-opioid drugs, BLA-dependent KOR-mediated analgesia offers the advantages of lower addiction potential and fewer side effects (Lonze and Ginty, 2002). This selective signal transduction mechanism paves the way for the development of novel analgesic drugs, particularly as replacements for or supplements to traditional opioids (Chavkin et al., 1982).

#### 2.2.2 Cross-mechanisms of morphine tolerance

The analgesic effect of BLA through the κ-opioid receptor pathway exhibits a complex cross-regulatory phenomenon with the μ-opioid receptor mechanism of morphine at the molecular level. Research (Li et al., 2010) has demonstrated that in a morphine-tolerant rat model, κ-opioid receptors are upregulated in the spinal cord and locus coeruleus, while they are downregulated in the dorsal root ganglion. Studies on molecular mechanisms (Yamada et al., 2006) have revealed a functional interaction between the κ-opioid receptor and the μ-opioid receptor.

Preclinical studies have further confirmed the synergistic effects of the combined application of BLA and morphine. Huang et al. demonstrated in various animal models of pain that BLA significantly enhance the analgesic effect of morphine while completely preventing the development of morphine tolerance (Huang et al., 2017). In neuropathic pain models, BLA not only inhibits formaldehyde-induced tonic pain but also produces additive analgesic effects in conjunction with morphine.

These findings hold significant clinical implications. By activating various opioid receptor subtypes and unique microglial regulatory mechanisms, BLA presents a novel therapeutic strategy for overcoming morphine tolerance. Particularly within multimodal analgesic regimens, BLA may function as an effective adjuvant to morphine, enhancing analgesic efficacy while minimizing the adverse effects associated with single-drug therapy.

### 2.3 Anti-inflammatory-immunomodulatory integrated effect

BLA, a natural diterpenoid alkaloid, exhibits a unique integrated mode of anti-inflammatory and immunomodulatory actions. This dual regulatory mechanism offers a novel approach for treating inflammatory diseases.

In terms of anti-inflammatory mechanisms, BLA significantly modulates the expression of pro-inflammatory factors. In a mouse model of allergic asthma (Zhan et al., 2019), BLA treatment notably reduced key pro-inflammatory factors, including IL-4, TNF-α, and MCP-1, in the bronchoalveolar lavage fluid. These factors are crucial mediators of allergic inflammatory responses and play a central regulatory role in the pathogenesis of asthma. More importantly, BLA simultaneously inhibits the NF-κB signaling pathway, which is pivotal in immunity, inflammation, and cell proliferation (Liu et al., 2017). This mechanism is significantly important for controlling inflammatory responses.

At the level of immunomodulation, BLA exhibits significant bidirectional regulatory characteristics. Research has demonstrated (Liu et al., 2020) that BLA suppresses airway hyperresponsiveness, pulmonary inflammation, and airway remodeling by restoring the Th1/Th2 balance. In an animal model of allergic asthma (Zhan et al., 2019), BLA not only significantly reduced the levels of total serum IgE and IgG but also decreased the counts of eosinophils, lymphocytes, and macrophages in the bronchoalveolar lavage fluid.

The integrated anti-inflammatory and immunomodulatory effects of BLA hold significant pathophysiological implications. In the context of autoimmune diseases, the overactivation of the immune system results in the release of numerous pro-inflammatory factors and abnormal immune cell functions, leading to tissue damage and chronic inflammation (Song et al., 2024). BLA restores the inflammation-immune balance at the molecular level through a unique dual regulatory mechanism that simultaneously inhibits the production of pro-inflammatory factors and modulates the functional state of immune cells.

### 2.4 Bone repair and tissue microenvironment regulation (emerging research focus)

Bone repair is a complex biological process that involves the precise coordination of multiple stages, including inflammation regulation, angiogenesis, osteoblast differentiation, and modulation of osteoclast activity (Bahney et al., 2019). Maintaining a balance between osteoblasts and osteoclasts is crucial for effective bone healing (Mi et al., 2022). Recent studies have revealed that BLA exhibits a unique dual regulatory mechanism in promoting bone repair: it not only inhibits excessive osteoclast activity but also optimizes the local tissue microenvironment, thereby creating favorable conditions for the osteogenic process.

Peng et al. (2023) established a mouse model of tibial fracture to validate the promoting effect of BLA on bone repair. The results demonstrated that BLA not only effectively alleviated mechanical and thermal hyperalgesia induced by the fracture, but, more importantly, significantly promoted fracture healing.

In terms of cellular molecular mechanisms, the study by Zhang et al. elucidated the inhibitory effect of BLA on osteoclast differentiation (Zhang et al., 2018). The research demonstrated that BLA inhibits the activation of the NF-κB signaling pathway, thereby obstructing the formation of osteoclasts and the activity of bone resorption. Building upon this foundation, Wang et al. (2024) further illustrated that, in addition to regulating osteoclasts, BLA downregulates the expression of pro-inflammatory factors such as TNF-α, IL-1, and IL-6, while also diminishing the production of prostaglandin E2 (PGE2). This modulation of inflammatory microenvironment at the molecular level occurs during the early stages of fracture.

This multi-target regulatory mechanism holds significant clinical translational value. During the early inflammatory phase of fracture healing, excessive inflammatory responses can delay the healing process and exacerbate pain (ElHawary et al., 2021). BLA precisely regulates the release of inflammatory factors, maintaining the necessary inflammatory response to initiate the repair process while preventing excessive inflammation that could lead to tissue damage. Simultaneously, its selective inhibition of osteoclast activity ensures that bone formation predominates during remodeling. This characteristic endows BLA with broad application prospects in the treatment of orthopedic diseases. More importantly, the bone repair-promoting effect of BLA, coupled with its analgesic properties, offers a novel solution and research direction for addressing the clinical challenge posed by traditional analgesic drugs, such as non-steroidal anti-inflammatory drugs, which may interfere with bone healing (Al Farii et al., 2021) (Table 2).

**TABLE 2:** Multi-target mechanism of action of the BLA.

Mechanism of action category	Key signaling pathway	Core effect	Clinical significance
Central microglia and the κ-opioid receptor pathway	Microglia in the spinal dorsal horn, κ-opioid receptor (KOR), PKA-p38 MAPK-CREB signaling cascade	① Induce microglia-specific release of dynorphin A (endogenous KOR agonist) ② Inhibit excitatory neurotransmitter release and enhance spinal inhibitory neuronal activity	Opening up new directions for non-μ-opioid analgesia, avoiding the risk of excessive immunosuppression, and replacing traditional opioid drugs
Cross-mechanisms of morphine tolerance	μ-opioid receptor (MOR), κ-opioid receptor (KOR), spinal microglia (pro-inflammatory factor release)	① Upregulation of spinal cord/locus coeruleus KOR, antagonizing MOR desensitization/downregulation ② Enhancing the analgesic effect of morphine ③ Complete suppression of morphine tolerance	As an adjuvant to morphine, it reduces the need for dose escalation, decreases its side effects (such as dependence), and optimizes chronic pain management
Anti-inflammatory -Immunomodulatory Integrated Effect	NF-κB pathway, Th1/Th2 balance, pro-inflammatory factors (IL-4/TNF-α/MCP-1), immunoglobulins (IgE/IgG)	① Inhibit NF-κB activation and reduce the expression of pro-inflammatory factors ② Restore Th1/Th2 balance, reduce infiltration of immune cells such as eosinophils Bidirectional regulation of immune function	To provide a dual effect of “anti-inflammatory + immunomodulatory” for inflammatory/autoimmune diseases, avoiding the limitations of single anti-inflammatory drugs
Bone Repair and Regulation of Tissue Microenvironment	Osteoclasts (NF-κB/NFATc1 pathway), bone repair microenvironment, pro-inflammatory factors (TNF-α/IL-1/IL-6), PGE2	① Inhibit osteoclast differentiation and promote callus formation (increase BV/BV/TV) ② Regulate the inflammatory microenvironment in the early stage of fracture to avoid excessive inflammatory damage, forming a positive cycle of “analgesia - movement - bone repair”	Addressing the challenge of traditional analgesics interfering with bone healing, providing an integrated solution of pain relief and repair for orthopedic conditions such as fractures

## 3 The pharmacokinetics of BLA

Although BLA demonstrates unique pharmacological advantages in its analgesic mechanism, it still encounters significant pharmacokinetic challenges in clinical applications, including complex drug metabolism processes, poor *in vivo* stability, and low bioavailability. These issues severely limit the expansion of its therapeutic window and the enhancement of clinical safety. Therefore, a comprehensive understanding of the pharmacokinetic characteristics of BLA is crucial for optimizing its clinical application.

### 3.1 CYP450 metabolic mechanism and drug interaction risks

The cytochrome P450 (CYP450) enzyme system constitutes the core regulatory network for drug metabolism within the body (Klein and Zanger, 2013). By catalyzing oxidation, reduction, and hydrolysis reactions of drugs, it directly influences the blood concentration levels of these substances, thereby affecting their therapeutic efficacy and potential toxic reactions (Miksys and Tyndale, 2009). Among the various subtypes of the CYP450 enzyme system, CYP3A4 assumes a predominant role (Guengerich, 2008). As a structurally complex diterpenoid alkaloid, the metabolic transformation of BLA in the body heavily relies on the catalytic function of the CYP450 enzyme system. Consequently, any factors that influence the activity of CYP450 enzymes, such as drug interactions or environmental factors, may significantly alter the pharmacokinetic characteristics of BLA, thereby impacting the safety and efficacy of its clinical application.

Li et al. (2022) identified that BLA serves as a sensitive substrate and competitive inhibitor of CYP3A4, which may significantly contribute to its clinical adverse reactions. The inhibition of CYP3A4 activity by BLA impacts the metabolic clearance of other drugs, thereby increasing the risk of drug accumulation and toxicity.

Therefore, a comprehensive understanding of the CYP450 metabolic characteristics of BLA and the potential risks of drug interactions, along with the establishment of a personalized medication monitoring system, is crucial for enhancing the safety and efficacy of its clinical application.

### 3.2 Pharmacokinetic deficiencies (narrow therapeutic window, first-pass effect, short half-life)

#### 3.2.1 Narrow therapeutic window and safety challenges

The therapeutic window serves as a critical indicator for assessing drug safety (Bémeur et al., 2007). As an aconitum alkaloid, BLA demonstrates both cardiac and neurotoxic effects, and its therapeutic window is notably narrow. The cardiovascular toxicity of BLA primarily manifests as palpitations, arrhythmias—including ventricular tachycardia and atrial fibrillation—and, in severe cases, may lead to life-threatening cardiotoxicity characterized by hypotension and bradycardia (Wang et al., 2008; Wang et al., 2007). These cardiovascular adverse effects are dose-dependent and represent a significant safety concern that limits the clinical application of BLA. Similar to other aconitine-type alkaloids, BLA can induce cardiac arrhythmias by disrupting intracellular ion homeostasis, particularly affecting voltage-gated sodium and calcium channels (Sun et al., 2014). Therefore, electrocardiographic monitoring is essential during BLA administration, especially when higher doses or intravenous routes are employed (Li et al., 2022).

BLA has been utilized in China since 1985 for the management of chronic pain; however, the safety margin between its effective dose and toxic dose is relatively narrow (Wang et al., 2007). Safety evaluation studies (Yin et al., 2021), have demonstrated that BLA at a dosage of 0.14 mg/kg exhibits significant analgesic effects in both the hot plate test and the acetic acid writhing test. In subchronic toxicity studies, the no-observed-adverse-effect level (NOAEL) was identified as 0.25 mg/kg, while the lowest-observed-adverse-effect level (LOAEL) was determined to be 0.5 mg/kg.

The study conducted by Wang et al. (2007) further substantiates the characteristic of a narrow therapeutic window. In a rat model, a concentration of 0.125 mM BLA effectively provided cutaneous analgesia without significant systemic side effects. However, at an increased concentration of 0.25 mM, severe toxic symptoms manifested in the experimental animals. Furthermore, at a concentration of 0.5 mM, all experimental subjects (3/3) succumbed within the administration period (Wang et al., 2008).

This relatively narrow therapeutic window necessitates precise dosing control in clinical applications, as well as the establishment of an effective blood concentration monitoring system. Individual differences further complicate the therapeutic window issue. Due to the genetic diversity of CYP3A4 (Lamba et al., 2002), patients can exhibit several-fold differences in their response to the same dose of BLA (Ingelman-Sundberg, 2004). Individuals with fast metabolism may require higher doses to achieve effective blood concentrations, however, this correspondingly increases the risk of toxicity. Conversely, individuals with slow metabolism may experience drug accumulation and toxic reactions even at conventional doses (Burk and Wojnowski, 2004).

#### 3.2.2 First-pass effect and bioavailability

BLA, a natural compound with multiple pharmacological activities, including anti-inflammatory effects, demonstrates promising potential in the treatment of rheumatoid arthritis. However, the significant first-pass effect encountered after oral administration severely limits the drug’s bioavailability and clinical efficacy.

To investigate the underlying causes of the low bioavailability of BLA, Li et al. elucidated the specific mechanisms involved in this process (Li et al., 2022). Research indicates that following gastrointestinal absorption, BLA is transported to the liver via the portal vein system, where it undergoes metabolism into various metabolites within hepatocytes. The analgesic activity of these metabolites is significantly lower than that of the parent compound, leading to a substantial reduction in therapeutic efficacy.

To overcome the limitations of the first-pass effect, researchers have explored various optimization strategies for drug delivery routes. Wang et al. (2007) demonstrated that subcutaneous administration of BLA effectively bypasses the hepatic first-pass effect, allowing the drug to reach effective concentrations directly at the site of action. Although intravenous administration can completely avoid the first-pass effect, it carries higher safety risks due to the cardiotoxicity associated with BLA. Therefore, clinical application necessitates strict electrocardiographic monitoring (Chan, 2009), which somewhat limits the feasibility of its clinical promotion.

#### 3.2.3 Short half-life and dosing frequency issues

A short half-life represents another significant drawback in the pharmacokinetics of BLA. The plasma elimination half-life of BLA is relatively short (Weng et al., 2005), which hinders the drug’s ability to maintain a sustained effective concentration in the body. Consequently, a frequent dosing regimen is required to uphold therapeutic blood levels. This dosing strategy not only considerably increases the medication burden and treatment costs for patients but also elevates the risk of drug accumulation and cumulative toxic reactions.

The existence of these pharmacokinetic challenges underscores the importance of developing BLA drug formulation technologies. Innovations in drug formulation are expected to address the limitations of BLA regarding stability, bioavailability, and ease of administration, ultimately offering a safer and more effective analgesic treatment option for clinical practice.

## 4 Novel drug delivery system

In recent years, researchers have developed various novel drug delivery systems that have improved the pharmacokinetic properties of BLA. These advancements have resulted in a prolonged duration of action, enhanced bioavailability, and reduced toxic side effects, thereby offering new possibilities for precise treatment.

### 4.1 Sustained-release microsphere formulation

The microsphere drug delivery system exhibits significant potential for the local sustained-release administration of BLA. Wang et al. (2024) developed long-acting BLA microspheres (BLA-MS) for intra-articular injection, successfully controlling the initial burst release to an exceptionally low level. This characteristic of low burst release effectively mitigates toxic side effects caused by high local concentrations and significantly extends the drug’s retention time within the joint cavity.

In the collagen-induced arthritis rat model, the localized sustained-release system of BLA-MS establishes a drug reservoir at the lesion site. This approach mitigates the risks of inflammatory damage and infection associated with frequent dosing, thereby significantly enhancing patient compliance.

### 4.2 Liposome drug delivery system

Liposomes are biocompatible nanocarriers that exhibit excellent properties, including the ability to prolong drug action time through sustained-release mechanisms and to reduce toxic reactions (Allen and Cullis, 2013). More importantly, liposomal technology can facilitate targeted delivery via surface modification, thereby expanding the clinical application possibilities of BLA (Sercombe et al., 2015).

The long-acting local anesthetic bupivacaine (Exparel) provides analgesic effects lasting up to 96 h through the use of liposome technology (Hussain et al., 2021). Consequently, BLA is anticipated to enhance efficacy and improve safety through the application of this technology.

### 4.3 ROS-responsive targeted delivery system

The application of nanocarrier technology has demonstrated revolutionary advantages in enhancing the targeting and bioavailability of BLA. Smart nanocarriers facilitate the selective enrichment of drugs at the lesion site while simultaneously minimizing toxic side effects on normal tissues (Mitchell et al., 2021). Li et al. (2025) developed reactive oxygen species (ROS)-responsive hierarchical targeting micelles (TK-FA-BLA-MS), which construct dual-responsive smart nanocarriers by integrating ROS-sensitive thioketal (TK) units and M1 macrophage-specific targeting groups, such as folic acid (FA).

The immunomodulatory effect achieved through the regulation of macrophage phenotypes unveils a novel mechanism of action for BLA in the treatment of rheumatoid arthritis, transcending its traditional analgesic and anti-inflammatory functions. This effect enhances the uptake efficiency of activated macrophages and promotes their polarization, thereby establishing a groundbreaking mechanism of action for BLA in the management of rheumatoid arthritis (Zhang et al., 2017).

## 5 Exploration of structural modification and toxicity separation

The narrow therapeutic window of BLA has significantly restricted its clinical application. In recent years, advancements in our understanding of BLA’s molecular mechanisms, coupled with rapid progress in structural biology (Jiang et al., 2022), computational chemistry (Tonoyan and Siraki, 2024), and artificial intelligence technologies (Zhang et al., 2025), have facilitated the transition of the concept of “toxicity-efficacy separation” from a theoretical idea to a practical approach.

**FIGURE 3:**
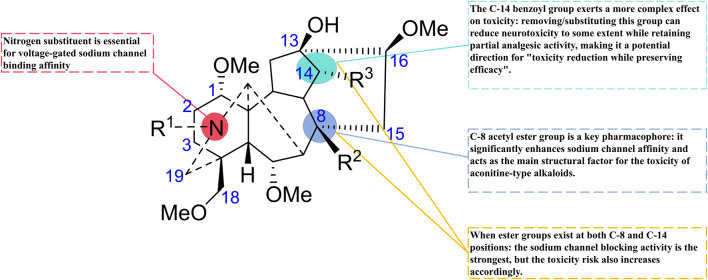
Key pharmacological and toxicological structural sites of BLA.

Structural modifications focus on selectively altering structural fragments that are closely related to toxicity but contribute relatively little to efficacy. Research has shown that while both the analgesic effect and neurotoxicity of BLA arise from its modulation of voltage-gated sodium channels, there are subtle differences in aspects such as binding sites. This provides a theoretical basis for achieving a separation of toxicity and efficacy through structural modifications. The philosophy of modern drug design aims to maintain or enhance analgesic activity while minimizing toxic side effects, employing systematic structure-activity relationship studies, innovative synthesis strategies, and intelligent molecular design (Guha, 2013) (Figure 3).

### 5.1 The relationship between essential groups and neurotoxicity

As a C19-type diterpenoid alkaloid, the acetyl ester group at the C-8 position and the benzoyl group at the C-14 position in the molecular structure of BLA are crucial for its pharmacological activity and toxicity. Structure-activity relationship studies (Ameri, 1998) have demonstrated that the ester substitutions at the C-8 and C-14 positions are the primary pharmacophores influencing its sodium ion channel binding activity. Modifications at these two sites directly impact the strength of the drug’s interaction with voltage-gated sodium ion channels (VGSCs), thereby determining the balance between its analgesic effects and neurotoxicity (Wang et al., 2010).

The presence of the C-8 acetyl ester group significantly enhances the compound’s affinity for sodium ion channels (Ameri, 1998), a structural feature that plays a dominant role in the toxic manifestations of aconitine-type alkaloids. Hydrolysis or substitution of the acetyl group at the C-8 position markedly reduces the acute toxicity of the compound; however, this alteration also weakens its analgesic activity (Li et al., 2016). In contrast, modifications to the C-14 benzoyl group exhibit a more complex influence on toxicity. Research indicates (Zhao et al., 2024) that the removal or substitution of the benzoyl group at the C-14 position can diminish neurotoxicity to some extent while preserving partial analgesic activity, thus providing a potential pathway for achieving a separation between toxicity and efficacy.

Moreover, a synergistic effect exists between the substituents at the C-8 and C-14 positions. When ester groups are present at both locations simultaneously, the compound demonstrates the strongest sodium channel blocking activity, albeit with a concomitant increase in toxicity risk (Bello‐Ramírez and Nava‐Ocampo, 2004). This discovery of the structure-activity-toxicity relationship has established a theoretical foundation for subsequent directed structural optimization (Table 3).

**TABLE 3:** Studies related to the synthesis of analogues and derivatives.

Modification site	New compound	Activity data	Structure-activity relationship	Reference
N	Imine compounds, lactam compounds, N-deethylated compounds	N-desethyl compound: ED_50_ = 0.411 mg/kg, 90% inhibition rate at 0.80 mg/kg dose, activity superior to the parent BLA; imine compound: ED_50_ = 6.42 mg/kg, reduced activity; lactam compound: 25.0% inhibition rate at 10 mg/kg dose, reduced activity	N-Deethylation significantly enhances analgesic activity, while imination or lactamization leads to a decrease in activity	Wang, (2004)
C-8 etherification	C-8 ethoxy compound; C-8 isopentyloxy derivative	C-8 ethoxy compound: ED_50_ = 0.0972 mg/kg, with an inhibition rate of 86.4% at a dose of 0.2 mg/kg, demonstrating excellent activity; C-8 isopentyloxy derivative: with an inhibition rate of only 15.0% at a dose of 10 mg/kg, showing poor activity	Small group etherification is beneficial for maintaining high activity, while bulky alkoxy substituents are detrimental to activity retention; the C-8 position is primarily associated with anti-inflammatory activity	(Wang et al., 2009) Mori et al. (1989), Kataria et al. (2024)
C-8 esterification	C-8/C-13 position diesterification product; C-14 position monoesterification product	Unspecified	​	Lin and Song (2021)
C-14 esterification	C-14 position monoester derivatives; amide benzoate derivatives; C-8 position acetylated diesters; C-8/C-13 position diacetylated triesters	At a dose of 10 mg/kg, the pain thresholds of six compounds increased by more than 100%; 5-chloro-2-thiophenecarboxylate was the most effective: the pain threshold increased by 166.35% at 15 min, reached 182.35% at 30 min (peak), and maintained 82.59% at 60 min, significantly outperforming BLA.	The C-14 position is closely related to analgesic activity and toxicity; modification with heterocyclic and polycyclic aromatic groups can enhance activity and prolong the duration of action; single ester modification at the C-14 position is more conducive to maintaining activity	(Lin and Song, 2021) Zhang et al. (2020), Cai et al. (2013)
C-14 etherification	C-14 position mono-substituted ethoxy product; C-8/C-14 position di-substituted product	Unspecified	To provide alternative strategies for constructing the ether bond at the C-14 position	Mori et al. (1989)

### 5.2 Advances in the synthesis of analogues and derivatives

In recent years, researchers have synthesized a series of BLA derivatives through structural modifications, aiming to reduce toxicity while maintaining or enhancing analgesic activity.

#### 5.2.1 Nitrogen atom modification

**FIGURE 4:**
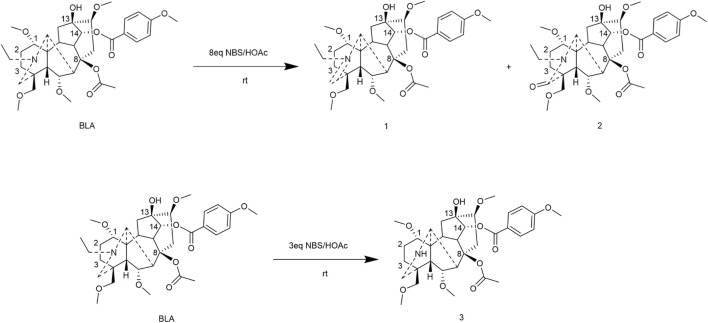
Synthesis of target compounds 1-3.

Wang (2004) first conducted a systematic study on the chemical modification of the nitrogen atom in BLA by regulating the amount of NBS/HOAc used for this modification. The study revealed that utilizing 8 equivalents of NBS/HOAc at room temperature resulted in the formation of an imine compound with a yield of 73%. Furthermore, extending the reaction time led to the production of lactam products. Conversely, reducing the amount of NBS to 3 equivalents produced N-deethylated compounds with a yield of 90% (Figure 4).

The evaluation of analgesic activity demonstrated that the N-deethylated compound exhibited significantly greater analgesic efficacy (ED_50_ = 0.411 mg/kg) compared to the parent BLA, achieving an analgesic inhibition rate of 90% at a dosage of 0.80 mg/kg. In contrast, the imine compound (ED_50_ = 6.42 mg/kg) and the lactam compound (with an inhibition rate of 25.0% at 10 mg/kg) displayed markedly reduced analgesic activity. Furthermore, the structure-activity relationship analysis revealed that the nature of the substituent on the nitrogen atom of the A ring plays a crucial role in influencing the analgesic activity of these compounds.

#### 5.2.2 C-8 position structural modification

##### 5.2.2.1 Etherification reaction

**FIGURE 5:**
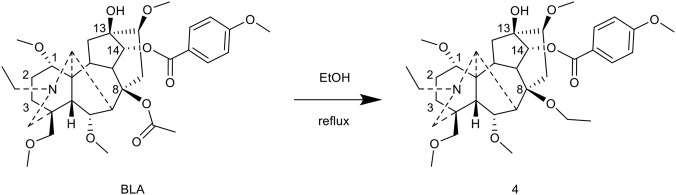
Synthesis of target compounds 4.

Wang et al. (2009) developed a method for the C-8 modification of BLA by adapting the etherification conditions used for norditerpenoid alkaloids. Refluxing BLA in ethanol for 48 h resulted in the formation of the C-8 ethoxy compound, which was obtained with an impressive yield of 89%. This compound demonstrated significant analgesic activity, with an ED_50_ of 0.0972 mg/kg and an analgesic inhibition rate of 86.4% at a dose of 0.2 mg/kg (Figure 5).

**FIGURE 6:**
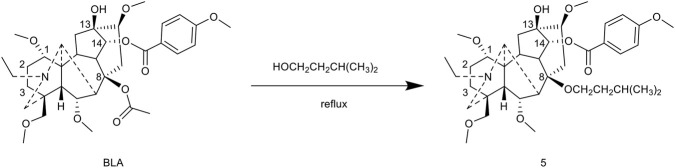
Synthesis of target compounds 5.

Using isopentanol as the reaction medium, the reaction was conducted at 80 °C for 3 h, resulting in the formation of the C-8 isopentoxy derivative 6 with a yield of 30%. However, the analgesic inhibition rate of this compound was only 15.0% at a dosage of 10 mg/kg, suggesting that larger alkoxy substituents adversely affect the retention of activity (Mori et al., 1989) (Figure 6).

##### 5.2.2.2 Esterification reaction

**FIGURE 7:**
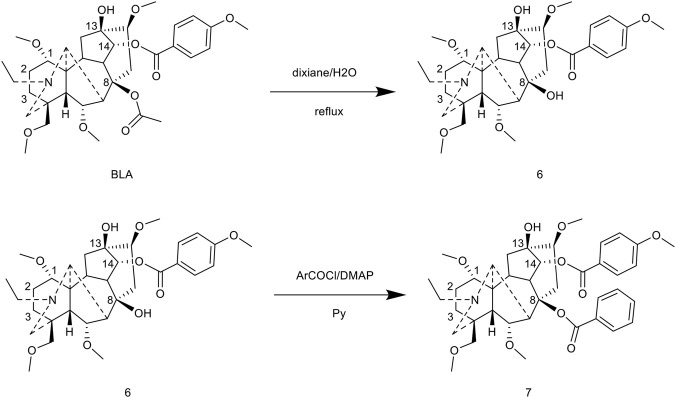
Synthesis of target compounds 6-7.

Lin and Song (2021) accomplished the esterification modification at the C-8 position via a multi-step synthesis. Initially, BLA was refluxed in a dioxane-water (1:1) mixed solvent to obtain the C-8 hydroxyl compound 6. This compound was subsequently esterified by reacting it with aryl acyl chloride in the presence of DMAP as a catalyst, using pyridine as the solvent and refluxing for 12 h, which yielded compound 7. The study revealed that both the solvent and temperature significantly influenced regioselectivity: refluxing in pyridine predominantly resulted in C-8/C-13 bis-esterified products, whereas the reaction conducted at room temperature in dichloromethane primarily yielded C-14 mono-esterified products (Figure 7).

#### 5.2.3 C-14 position structural modification

##### 5.2.3.1 Esterification reaction

Zhang et al. (2020) conducted the most comprehensive study to date on C-14 position modification. They began with the complete hydrolysis product obtained from the alkaline hydrolysis of BLA and reacted it with 27 different acyl chlorides in a pyridine/dichloromethane system at 40 °C for 2 h. This process resulted in the synthesis of a series of C-14 monoester derivatives, with yields ranging from 25% to 70%. The derivatives included aliphatic, aromatic, and heterocyclic carboxylate esters.

**FIGURE 8:**
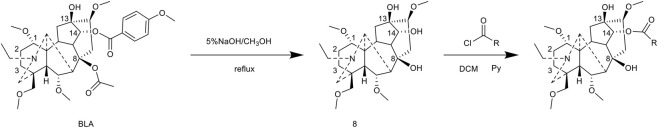
Synthesis of target compounds 8.

The hot plate analgesic test (10 mg/kg) revealed that six compounds exhibited remarkable analgesic activity, with pain threshold increases exceeding 100%. Notably, 5-chloro-2-thiophenecarboxylate demonstrated the most significant performance: at 15 min, the pain threshold increased by 166.35%, peaked at 182.35% at 30 min, and maintained a level of 82.59% at 60 min. This performance significantly surpassed that of BLA, which resulted in a pain threshold increase of only 69.60% at 60 min with a dosage of 0.35 mg/kg (Figure 8; Table 4).

**TABLE 4:** Synthesis of target compounds 8a-8w.

Compound	8a	8b	8c	8d	8e	8f	8g	8h
R	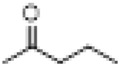	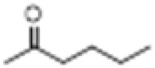	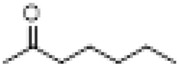	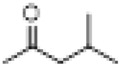	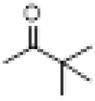	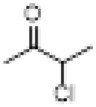	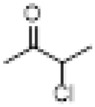	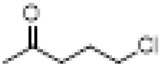
Compound	8i	8j	8k	8l	8m	8n	8o	8p
R	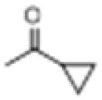	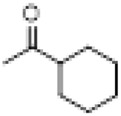	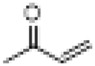	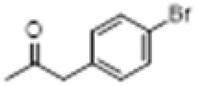	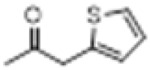	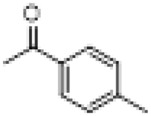	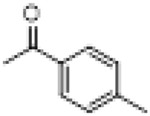	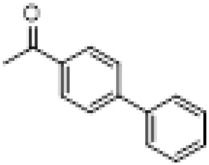
Compound	8q	8r	8s	8t	8u	8v	8w	​
R	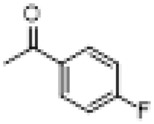	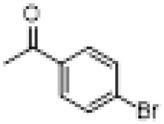	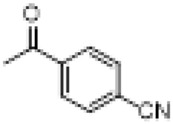	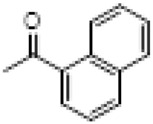	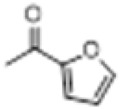	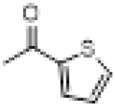	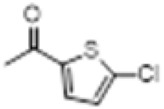	​

##### 5.2.3.2 Amidobenzoate derivatives

**FIGURE 9:**
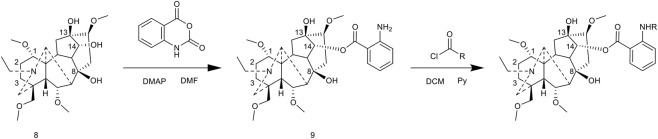
Synthesis of target compound 9.

Inspired by the modification of lappaconitine, Zhang et al. (2020) designed and synthesized a series of 2′-acylamino benzoates. The complete hydrolysis product reacted with phthalic anhydride in DMF at 120 °C for 7 h under DMAP catalysis, yielding the 2′-aminobenzoate intermediate with a yield of 63%. This intermediate was subsequently acylated to obtain five derivatives, with yields ranging from 30% to 59%. Although the analgesic activity of this series of compounds was slightly inferior to that of heterocyclic esters, it provided a structural basis for exploring the influence of hydrogen bonding on activity (Figure 9; Table 5).

**TABLE 5:** Synthesis of target compounds 9a-9e.

Compound	9a	9b	9c	9d	9e
R	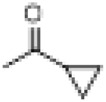	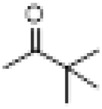	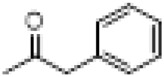	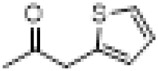	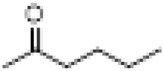

##### 5.2.3.3 Polyesterification research

**FIGURE 10:**
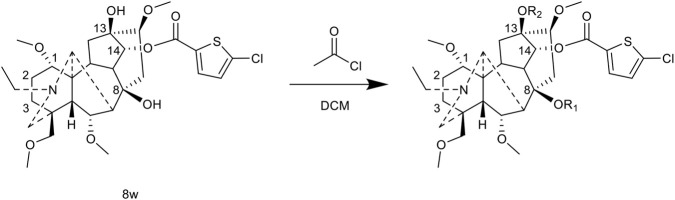
Synthesis of ester derivatives.

To validate the assertion in the literature that “diester diterpenoid alkaloids exhibit stronger activity” (Cai et al., 2013), Zhang et al. reacted the optimal monoester compound (5-chloro-2-thiophenecarboxylate) with acryloyl chloride at 35 °C for 30 min, yielding the C-8 acetylated diester (35% yield) and the C-8/C-13 diacetylated triester (61% yield). Unexpectedly, the analgesic activity of the polyester compounds was significantly lower than that of the monoester parent, indicating that for BLA, monoester modification at the C-14 position is more conducive to maintaining activity (Figure 10; Table 6).

**TABLE 6:** Synthesis of target compounds 8w_1_-8w_2_.

Compound	8w_1_	8w_2_
R_1_	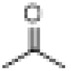	H
R_2_	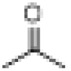	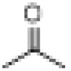

##### 5.2.3.4 Etherification reaction

**FIGURE 11:**
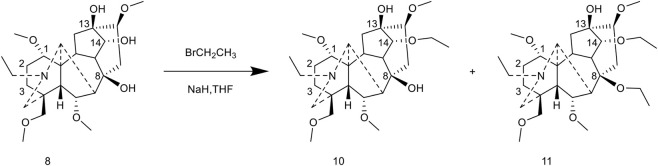
Synthesis of target compounds 10-11.

The Wang group (Mori et al., 1989) reported an etherification modification at the C-14 position. The complete hydrolysis product was reacted with sodium hydride (NaH) in tetrahydrofuran (THF) for 2 h, followed by the slow addition of bromoethane and a continued reaction for 4 h. This process yielded the C-14 ethoxy mono-substituted and C-8/C-14 di-substituted products. This method offers an alternative strategy for constructing ether bonds; however, the activity data of the resulting products have not been fully reported (Figure 11).

Through systematic structural modifications at multiple sites of BLA, the researchers synthesized a series of novel derivatives that exhibit improved pharmacological properties. These compounds not only retained or enhanced the analgesic activity of the parent drug but also significantly reduced toxicity. Moreover, some compounds demonstrated additional therapeutic potentials, including anti-inflammatory and anxiolytic effects. Future research should continue to explore the structure-activity relationships to develop a new generation of safer and more effective analgesic drugs.

### 5.3 AI-assisted structure-activity relationship prediction

With the advancement of computational chemistry and artificial intelligence technologies, quantitative structure-activity relationship (QSAR) analysis, along with molecular docking techniques based on the BLA molecular structure, has provided theoretical guidance for research on the separation of toxicity and efficacy.

The BLA QSAR study has established a quantitative relationship between its structural characteristics and toxicity. Koleva et al. demonstrated that compounds possessing an aroyl/aroyloxy group at the C-14 position (such as BLA and aconitine) exhibit greater toxicity compared to those with an aroyloxy group at the C-4 position (Bello‐Ramírez and Nava‐Ocampo, 2004). Moreover, machine learning algorithms can develop QSAR models based on features such as molecular weight and partition coefficient, facilitating accurate predictions of BLA’s cardiac and neurotoxicity (Shah et al., 2024).

Furthermore, molecular docking technology can be employed to predict the binding modes and affinities of BLA and its derivatives with voltage-gated sodium ion channels. This approach enables the evaluation of the effects of C-8 acetyl ester and C-14 benzoyl modifications on the strength of protein-ligand binding.

By integrating various methods such as QSAR, molecular dynamics simulations, and free energy calculations (Turabekova et al., 2008), this study aims to provide systematic guidance for the separation of toxicity and efficacy in biological ligand activity (BLA) and to facilitate rational drug design.

### 5.4 Technology-driven BLA optimization

Biologics, as traditional natural products, face numerous challenges in modern clinical applications; however, technological innovations have opened broad prospects for their future development. Artificial intelligence is revolutionizing drug development models by employing machine learning algorithms to predict toxicity profiles and optimize compound structures, thereby providing new approaches for developing safer biologic derivatives (Fu and Chen, 2025). The intelligent design of novel drug delivery systems facilitates the precise development of targeted drug delivery and controlled-release formulations, which hold promise for addressing the bioavailability and targeting issues associated with biologics (Serrano et al., 2024).

The synergistic development of these technological innovations will facilitate the transformation of BLA from traditional analgesic drugs to individualized precision medicine, thereby playing a more significant role in the field of pain medicine.

## 6 Clinical translation and new indication prospects

With the in-depth development of modern pharmacological research, BLA has emerged as a significant drug in the field of pain management, exhibiting a unique non-opioid receptor-dependent analgesic mechanism. This characteristic enhances its potential for clinical application, particularly in light of the ongoing opioid abuse crisis.

### 6.1 Re-evaluation of efficacy in traditional indications (osteoarthritis, cancer pain, neuropathic pain, etc.)

BLA has received approval in China for the treatment of chronic pain and rheumatoid arthritis (Xie et al., 2018b). Recent clinical and basic research has further substantiated its traditional indications.

In the treatment of neuropathic pain, clinical studies have demonstrated that BLA can effectively alleviate human neuropathic pain (Wang et al., 2007). Furthermore, BLA has been shown to mitigate paclitaxel-induced neuropathic pain (Zhu et al., 2015).

In the treatment of arthritis, BLA demonstrates anti-inflammatory and analgesic effects by decreasing the expression of prostaglandin E2 (PGE2). Recent studies have developed long-acting BLA microspheres, which offer multidimensional therapy for rheumatoid arthritis via intra-articular administration, thereby showcasing their potential for localized drug delivery (Wang et al., 2024).

In the context of cancer pain, BLA has been shown to effectively inhibit both peripheral and central sensitization associated with chronic pain; however, it does not exert any influence on acute pain (Xie et al., 2018b).

In summary, BLA does not induce tolerance or addiction in the treatment of chronic pain. However, to further optimize its application in traditional indications, it is necessary to conduct more high-quality randomized controlled trials to verify its long-term efficacy and safety, as well as to establish standardized dosing regimens and monitoring indicators (Li et al., 2022).

### 6.2 The advantages of combination therapy with opioids

Current clinical guidelines for pain management increasingly emphasize the significance of multimodal analgesia. This approach minimizes adverse effects by employing medications with diverse mechanisms of action. A notable advantage of combining BLA in therapy is its capacity to diminish opioid tolerance. Research indicates (Mai et al., 2020) that BLA’s unique analgesic mechanism, which operates independently of opioid receptors, not only delivers effective pain relief but also mitigates opioid tolerance and dependence. This offers new avenues for developing safer and more effective pain management strategies (Zhu et al., 2015) (Table 7).

**TABLE 7:** Comparison of key pharmaceutical and clinical characteristics between BLA and morphine.

Parameter	BLA	Morphine
Efficacy	Hot plate test and acetic acid writhing test: 0.14 mg/kg (Yin et al., 2021)	Tail-flick test ED_50_: 3.21 mg/kg (intravenous injection)(Niemegeers et al., 1976)
LD_50_	Mice Subcutaneous injection: 0.92 mg/kg (Xiong et al., 2009) Rats Subcutaneous injection: 0.51 mg/kg Oral administration: 3.4434 mg/kg (Yin et al., 2021)	Mice Subcutaneous injection: 670 mg/kg (Funahashi et al., 1988) Intraperitoneal injection: 400 mg/kg (Grant et al., 1994) Wild-type mice: approximately 500 mg/kg (Sora et al., 2001) Rats Safety margin for intravenous injection: 1:69.5 (Niemegeers et al., 1976)
MTD	Rats LOAEL (Lowest Observed Adverse Effect Level): 0.5 mg/kg (oral) NOAEL (No Observed Adverse Effect Level): 0.25 mg/kg (oral) (Yin et al., 2021)	Rats Intravenous injection: approximately 100 mg/kg (significant interspecies variation)(Strandberg et al., 2006)
Clinical Dosage	Intramuscular injection: 0.2 mg/2 mL (Wang et al., 2007) Oral tablet: 0.4 mg/tablet (Wang et al., 2007)	The clinical dosage range is wide, and adjustments are required based on patient tolerance and pain intensity (Dowell et al., 2016)
t_1_/_2_	After 0.2 mg intramuscular injection: 4.88 ± 0.97 h (Weng et al., 2005)	After intravenous injection: 2–3 h (Berkowitz, 1976; Stanski et al., 1978) Terminal half-life after oral administration: 15.1 ± 6.5 h (due to enterohepatic circulation) (Hasselström and Säwe, 1993)
Addictiveness	Non-addictive (Tang et al., 1986)	Highly addictive (Darcq and Kieffer, 2018)
Gastrointestinal adverse reactions	Fewer than morphine (Tang et al., 1986)	Common nausea, vomiting, constipation, and abdominal distension; constipation persists with long-term use (Benyamin et al., 2008)
Neurotoxicity	Potential neurotoxicity at concentrations >0.25 mM, but low risk within therapeutic window; toxicity separable from efficacy through structural modifications (Wang et al., 2008)	Chronic use may exacerbate neuropathic pain and cause cognitive impairment (Mao, 2002)
Tolerance	No tolerance development (Tang et al., 1986; Huang et al., 2017)	Prone to tolerance with chronic use, requiring progressive dose escalation to maintain equivalent analgesic effects (Collett, 1998)
Mechanism of action	Multi-targeted: state-dependent blockade of voltage-gated sodium channels, activation of spinal microglial κ-opioid receptor pathway, anti-inflammatory immunomodulation, promotion of bone repair	Primarily dependent on μ-opioid receptor activation, inhibiting excitatory neurotransmitter release and blocking nociceptive transmission (Waldhoer et al., 2004)

### 6.3 New potential areas

#### 6.3.1 Visceral pain, IBS

Visceral pain is the primary symptom of irritable bowel syndrome (IBS), affecting approximately 10%–20% of the global population. The efficacy of traditional pharmacological therapies for IBS-related visceral pain is often suboptimal, underscoring the necessity for exploring new therapeutic targets and medications. BLA has demonstrated significant efficacy in treating chronic visceral hypersensitivity. In models of visceral pain induced by colonic inflammation, BLA effectively alleviates visceral pain responses and significantly improves visceral hypersensitivity (Huang et al., 2020a). Moreover, BLA is less likely to induce tolerance and addiction when alleviating visceral pain, thus providing a safer option for the long-term management of IBS-related visceral pain.

#### 6.3.2 Adjuvant therapy for neuropsychiatric disorders (anti-anxiety)

Chronic pain frequently coexists with psychiatric symptoms, including anxiety and depression, which significantly diminish patients’ quality of life. Preclinical studies have demonstrated that BLA exhibits dual efficacy in the treatment of pain and anxiety. In animal models of chronic visceral hypersensitivity and comorbid anxiety (Huang et al., 2020b), BLA was shown to alleviate pain while simultaneously improving anxiety-like behaviors. Compared to traditional combination therapy approaches, BLA offers both analgesic and anxiolytic effects, which may enhance patient compliance (Li et al., 2025).

## 7 Conclusion

Chronic pain is a significant global health issue that necessitates the urgent development of safe and effective treatment options (Gregory et al., 2013; Xie et al., 2018b). Current treatment methods are limited by issues such as addiction and gastrointestinal adverse reactions (Baron et al., 2010). BLA, a diterpenoid alkaloid derived from plants in the genus Aconitum of the Ranunculaceae family, exerts analgesic effects through multiple mechanisms. These include a state-dependent blockade of voltage-gated sodium channels, activation of the κ-opioid receptor pathway in spinal microglia, anti-inflammatory and immunomodulatory actions, and promotion of bone repair. BLA demonstrates low addiction potential and minimal adverse reactions (Tang et al., 1986), and has shown significant efficacy in various pain models, including neuropathic pain, cancer pain, and rheumatoid arthritis.

However, the clinical translation of BLA still faces significant challenges. Its narrow therapeutic window, low bioavailability, risk of drug interactions, and potential neurotoxicity limit its widespread application (Weng et al., 2005). To address these limitations, researchers have made groundbreaking progress in various directions in recent years. Novel drug delivery systems have significantly improved the pharmacokinetic characteristics of BLA, extending its duration of action and minimizing adverse effects. Structural modification studies have successfully synthesized a series of BLA derivatives that separate toxicity from efficacy. Additionally, QSAR analysis and molecular docking techniques have provided robust tools for the rational design of BLA, thereby accelerating the translation process from the laboratory to clinical settings.

In clinical applications, BLA has demonstrated not only good efficacy in traditional indications but also significant therapeutic potential in emerging fields such as visceral hypersensitivity, irritable bowel syndrome, and pain-related anxiety disorders. Notably, when combined with opioids, BLA significantly enhances analgesic effects and completely inhibits the development of morphine tolerance, thereby providing a novel therapeutic strategy for multimodal analgesia.

In summary, BLA, a non-opioid analgesic with multi-target therapeutic potential, presents significant prospects for application in the field of pain medicine (Xie et al., 2018b). Future research should capitalize on these technological advancements to develop novel drugs that exhibit higher selectivity and broader therapeutic windows (Li et al., 2017). Additionally, it is crucial to construct safer, more efficient, and precisely controllable intelligent drug delivery platforms. This approach will facilitate the transformation of BLA from a traditional analgesic into a personalized precision therapeutic agent, thereby laying a solid foundation for its further development and clinical application, and offering new directions for the treatment of chronic pain.
